# Thyroid Storm-induced Severe Dilated Cardiomyopathy and Ventricular Tachycardia

**DOI:** 10.7759/cureus.5079

**Published:** 2019-07-04

**Authors:** Kirolus Sourial, Saif M Borgan, Jorge E Mosquera, Loui Abdelghani, Aamir Javaid

**Affiliations:** 1 Internal Medicine, University of Central Florida College of Medicine/Hospital Corporation of America Graduate Medical Education Consortium, Orlando, USA; 2 Cardiology, University of Central Florida College of Medicine/Hospital Corporation of America Graduate Medical Education Consortium, Orlando, USA

**Keywords:** thyroid storm, graves’ disease, autoimmune myocarditis, non-ischemic cardiomyopathy.

## Abstract

Thyroid storm is an extreme form of hyperthyroidism associated with a high mortality rate. Heart failure is considered the leading cause of mortality in patients with thyroid storm, though the underlying cardiac pathology is unclear. Approximately 6% of patients with thyroid storm have heart failure symptoms as the initial presenting complaint. Roughly, one-third of these patients develop dilated cardiomyopathy (DCM). In this report, we present a case of cardiogenic pulmonary edema and sustained ventricular tachycardia in a patient with hyperthyroidism presenting with thyroid storm.

## Introduction

Cardiovascular manifestations of hyperthyroidism are related to decreased systemic vascular resistance and hyperdynamic status [1]. Hyperthyroidism is an uncommon cause of dilated cardiomyopathy (DCM) and heart failure. Burch-Wartofsky criteria are used in the diagnosis of thyroid storm [2]. If a patient develops cardiomyopathy without a known etiology, it is essential to assess the patient’s thyroid function, as early treatment of hyperthyroidism can reverse the left ventricular dysfunction [1]. In this report, we present a rare case of thyrotoxic cardiomyopathy (TCMP) with severe left ventricular dysfunction. We also discuss possible pathopysiology of how hyperthyroidism can cause cardiomyopathy and explore currently available strategies in the treatment of patients with hyperthyroidism and cardiomyopathy.

## Case presentation

**Figure 1:**
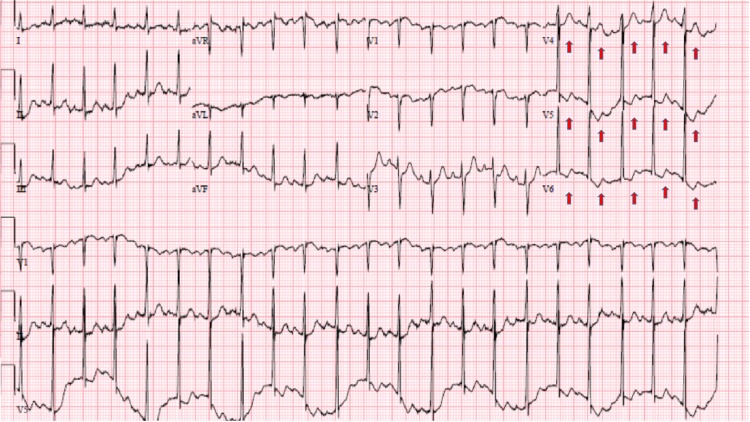
Electrocardiogram showing sinus tachycardia in addition to nonspecific ST and T wave abnormalities (red arrows) in leads V4- V6

A 52-year-old woman with a past medical history of uncontrolled Graves’ disease (GD) secondary to medication non-compliance presented to our emergency department with a one-week history of worsening dyspnea and increasing anxiety symptoms. Upon evaluation, the patient was noted to be in moderate respiratory distress, tachycardic, afebrile, anxious and tremulous. The rest of the physical exam was remarkable only for bilateral fine rales up to mid lung zones. Electrocardiogram showed sinus tachycardia with a heart rate of 133 beats per minute (BPM) and nonspecific ST and T wave changes (Figure 1).

**Figure 2:**
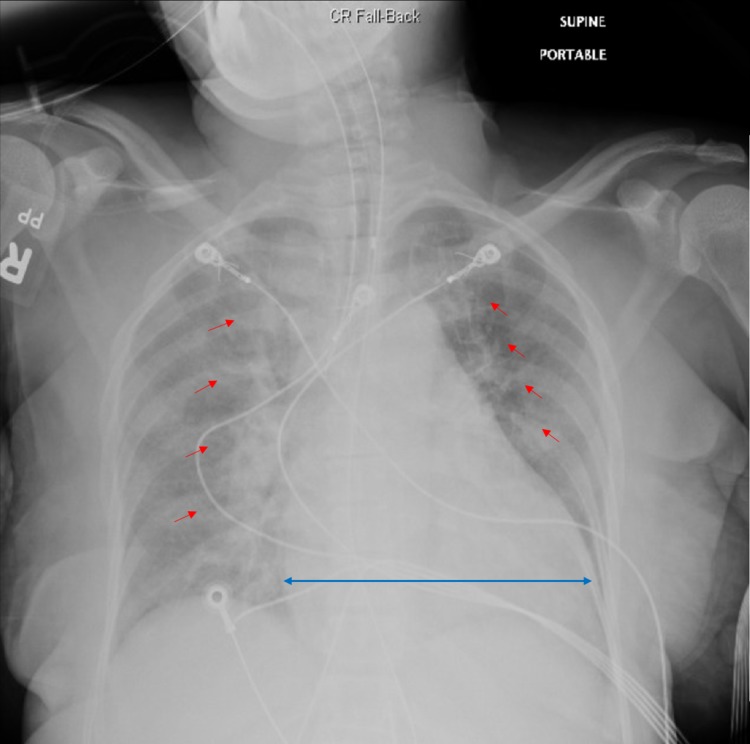
Post intubation chest X-ray showing increased bilateral airspace opacities which represent pulmonary edema (red arrows) and trace bilateral pleural effusions along with cardiomegaly (blue arrow)

Emergency measures included administration of Ativan and noninvasive respiratory support through bilevel positive airway pressure (BiPAP) resulted in minimal improvement. Blood test results showed extremely elevated free T3 and T4 levels >20.00 pg/mL and >8.00 ng/dL respectively, with suppressed thyroid-stimulating hormone (TSH) <0.01 uIU/mL and positive thyroid antibody levels with thyroid peroxidase antibody >1000.0 IU/ml. Initial Burch-Wartofsky score was 35. The patient developed flash pulmonary edema and sustained ventricular tachycardia. She required emergent cardioversion followed by endotracheal intubation and mechanical ventilation support. Post intubation chest X-ray confirmed the development of pulmonary edema (Figure 2).

Follow-up Burch-Wartofsky score was 55. Diuresis with intravenous Lasix was initiated along with propranolol, propylthiouracil, and corticosteroid therapy. Echocardiography was remarkable for severe DCM and severe global hypokinesis with an ejection fraction of 20% to 25%. The patient’s condition improved dramatically after 48 hours and she was successfully extubated. Her medical regimen was switched to oral carvedilol, tapering steroid dose, and methimazole. Prior to discharge, a pharmacological nuclear cardiac stress test ruled out the possibility of ischemic heart disease as an etiology of the cardiomyopathy.

The patient was provided with a life vest, and scheduled for follow up echocardiogram in three months.

## Discussion

Thyroid storm in the setting of GD often presents after a precipitating factor such as surgery, infection, non-compliance with anti-thyroid medication, or radioactive iodine ablation, with prior illness being the most common trigger [2]. Our patient reported non-adherence to her prescribed anti-thyroid medications due to financial hardship and inability to afford physician follow-up visits.

Burch-Wartofsky score is used to assess the probability of thyrotoxicosis based on clinical findings [2]. A score greater than 45 is highly suggestive of thyroid storm. Our patient had a score of 55 within hours of presentation. Refer to Table 1 for more information about Burch-Wartofsky score [3].

**Table 1:** Burch-Wartofsky score A score of < 25: less likely to be thyroid storm, 25-44: suggestive of thyroid storm, and > 44: highly suggestive of thyroid storm. C: celsius; BPM: beat per minute; CNS: central nervous system; GI: gastro-intestinal; N: nausea; V: vomiting.

Temperature	Points	Heart Rate	Points	Cardiac Manifestations	Points	CNS Manifestations	Points	GI manifestations	Points	Suggestive History	Points
< 37.7 °C	5	90 to 109 BPM	5	Lower extremities edema	5	Agitation	10	Diarrhea, N, V, or abdominal pain	10	Positive for suggestive History	10
37.8 to 38.3 °C	10	100 to 119 BPM	10	Bibasilar Lung rales	10	Delirium, Psychosis, or extreme lethargy	20	Unexplained Jaundice	20		
38.4 to 38.8 °C	15	120 to 129 BPM	15	Pulmonary Edema	15	Seizures, or Coma	30				
38.9 to 39.4 °C	20	130 to 139 BPM	20	Arrhythmia	10						
39.5 to 39.9 °C	25	> 140 BPM	25								
> 40 °C	30										

Elevated thyroid hormone levels are thought to mimic adrenergic excess state by directly upregulating cardiac β receptors and enhancing myocardial sensitivity to sympathetic and vagal innervation [2,4-5]. Thyroid hormone can also indirectly cause peripheral vasodilatation, decrease systemic vascular resistance, and increase in cardiac output by up to 300% [2]. Hence, beta-blockers are a vital component of thyroid storm management. In patients with thyroid storm and congestive heart failure, non-selective beta-blockers should be used carefully to decrease the risk of heart failure exacerbation [6].

Irregular cardiac rhythms are thought to be the main cardiac manifestation in patients with thyrotoxicosis. However, less frequent manifestations such as severe cardiac dysfunction should also be considered. For example, hyperthyroidism-induced Takotsubo cardiomyopathy has been previously reported [7].

Unfavorable prognostic factors in TCMP include the age of onset, atrial fibrillation during hospitalization, and recurrences of thyrotoxicosis. Electrocardiographic changes seen in TCMP, such as T wave inversions, are commonly seen at the time of presentation and can be easily attributed to ischemia, which could delay the diagnosis and management and lead to unfavorable outcomes [8].

Despite the known association between thyroid storm and cardiomyopathy, the underlying cardiac pathophysiology is still not completely understood [9]. GD is a systemic autoimmune disorder [10]. One study reported an autoimmune mechanism could be contributing to the myocardial dysfunction in TCMP [1]. Lymphocytic myocarditis has been described in an autopsy of a refractory case of congestive heart failure secondary to hyperthyroidism [9]. The previous data support the use of steroids in some cases of thyrotoxicosis. For example, one case report showed that a patient with thyroid storm-induced severe cardiomyopathy had a dramatic recovery after steroid pulse therapy. It is believed that the anti-inflammatory effect of steroids improves cardiomyopathy induced by thyroid storm [1]. However, there are no available guidelines regarding the optimal formulation, dose or duration of steroids.

Anti-thyroid drug (ATD) therapy (propylthiouracil and thiamazole) can also improve the recovery of the cardiomyopathy by suppressing the thyrotoxic state and correcting the primary hemodynamic disturbances [4]. In one study, seven thyrotoxic patients with congestive heart failure showed an increase of mean left ventricular ejection fraction (LVEF) from 28% to 55% after ATD therapy [11]. Early diagnosis of these rare and potentially reversible cases is important to improve the heart function and patient outcomes. The traditional management includes ATD to suppress thyroid hormone synthesis and release, and beta-blockers to protect myocardial tissues against the harmful effect of thyroid hormones. Steroids are recommended in life-threatening thyroid storm, but their use in managing non-storm TCMP is unclear.

## Conclusions

Early diagnosis of these rare and potentially reversible TCMP cases is important to improve the heart function and patient outcomes. Steroids use in managing non-storm TCMP is unclear. Further studies are needed to establish steroid effectiveness in lower-risk patients.
